# What (not) to eat: Exploring weight‐loss and dietary intentions in representative samples from Germany and Austria

**DOI:** 10.1111/aphw.70077

**Published:** 2025-09-13

**Authors:** Alea Ruf, Laura M. König

**Affiliations:** ^1^ Department of Clinical and Health Psychology Faculty of Psychology, University of Vienna Austria

**Keywords:** approach, avoidance, diet, eating, intention, weight loss

## Abstract

Despite significant health risks associated with high body weight and poor diet, little is known about the prevalence and targets of weight‐loss and dietary intentions. This information could, however, help tailor behaviour change interventions. Therefore, the present study described weight‐loss and dietary intentions and their co‐occurrence in a representative sample (*N* = 1,510; 50.40% women; *M*_*age*_ = 48.55, *SD*_*age*_ = 16.68; *M*_*BMI*_ = 26.89, *SD*_*BMI*_ = 5.92) from Germany (*n* = 1,006) and Austria (*n* = 504). Weight‐loss (57%) and dietary avoidance intentions (i.e., intention to eat less of certain foods; 59%) were more prevalent than dietary approach intentions (i.e., intention to eat more of certain foods; 34%). A discrepancy between weight‐loss intention and indication (i.e., meeting BMI criteria for weight‐loss recommendation: BMI ≥ 25) was found for 27% of individuals. Most common target foods were ‘snacks high in sugar, fat, and/or salt’ (24%), ‘meat’ (12%) and ‘sugar/foods high in sugar’ (11%) for avoidance and ‘fruits and vegetables’ (27%) and ‘protein/foods high in protein’ (3%) for approach intentions. These findings indicate that individuals might benefit from enhanced awareness of body weight recommendations and a less avoidance‐centered perspective on eating, as approach strategies might be more effective in changing behaviour.

## INTRODUCTION

High body weight and poor diet pose a significant threat to human health (Afshin et al., 2019; Zhou et al., 2024). They account, together with tobacco use and lack of physical activity, for more than half of all premature deaths from non‐communicable diseases (World Obesity Federation, 2025). Dietary risk factors associated with premature death from non‐communicable diseases include high intake of sodium, processed meat and sugar‐sweetened beverages as well as low intake of whole grains, fruit and vegetables (World Obesity Federation, 2025). With the United Nations likely not achieving their Sustainable Development Goals' target 3.4 aiming to reduce premature mortality from non‐communicable diseases by one third by 2030 (World Obesity Federation, 2025), promoting behaviour change and thereby weight loss as well as healthy eating remains a global challenge.

### Intentions to tailor behaviour change interventions

According to many health behaviour models, forming the intention to lose weight or eat healthy is an essential step towards weight loss and a healthy diet, as they understand intentions as the most important predictor of behaviour, e.g., theory of planned behaviour (Ajzen, 1991). Yet, intentions are not always translated into actual behaviour, known as the intention‐behaviour gap (Sheeran, 2002). Hence, interventions are needed to support individuals in translating their intentions into behaviour. So far, little is known about weight‐loss intentions (i.e., intention to reduce one's body weight) and dietary intentions (i.e., intention to eat more/less of certain foods), such as who intends to lose weight, or what individuals intend (not) to eat. However, this information could help tailor behaviour change interventions which has been shown to have positive effects on their uptake, engagement and effectiveness (Eyles & Mhurchu, 2009; König et al., 2021; Kroeze et al., 2006). For instance, a meta‐analysis found that tailored nutrition education is more effective in improving dietary intake compared to generic nutrition education (Eyles & Mhurchu, 2009). Beyond that, König et al. highlight the importance of tailoring nutrition app content to the user's goals, expectations and needs in order to increase uptake and engagement (König et al., 2021). Knowing how many and which individuals have weight‐loss and/or dietary intentions can help identify intervention targets as well as target groups that should be addressed. Hence, taking individuals' intentions into account when developing and tailoring weight‐loss and dietary interventions has the potential to improve uptake, increase engagement and achieve better outcomes.

#### Weight‐loss intentions

Several studies from the United States provide estimates on the prevalence of weight‐loss intentions. For instance, an increasing trend in the prevalence of weight‐loss intentions in adults from 62% in 1999–2000 to 67% in 2015–2016 was reported (Han et al., 2019). A systematic review found prevalences of weight‐loss intentions in youth in the US and Canada between 27% and 61% (Houle‐Johnson & Kakinami, 2018). Beyond that, from middle school onwards, girls were more likely to have weight‐loss intentions than boys. Based on these findings, the authors highlight that demographic characteristics should be considered when reporting prevalences for weight‐loss intentions, as specific groups might need targeted public health interventions (Houle‐Johnson & Kakinami, 2018). The need for group‐targeted interventions becomes also evident in a sample of adults aged 70 to 79 years, in which individuals with weight‐loss intentions (27% of the sample) were more likely to be female, have graduated from high school, and have higher body weight (Lee et al., 2004).

#### Dietary intentions

Given that health behaviours include approach behaviours (i.e., doing something) as well as avoidance behaviours (i.e., not doing something) (Dombrowski & Molloy, 2025), dietary intentions can be based on two strategies: (1) intention to eat foods that one should eat (i.e., dietary approach intention), and (2) intention not to eat foods that one should not eat (i.e., dietary avoidance intention) (Buhrau, 2020; David & Haws, 2016). While some research indicates that approach strategies seem generally more effective in improving diet quality (David & Haws, 2016), other research has shown that the weight status of individuals should be taken into account when choosing approach and avoidance strategies. Individuals with poor weight status (i.e., large difference between current and ideal weight) might benefit from approach strategies, as they motivate goal‐consistent behaviours by increasing the perceived attainability of the goal (Buhrau, 2020). In contrast, avoidance strategies might be more effective for individuals with good weight status (i.e., small differences between current und ideal weight), as they decrease perceived goal progress and thereby increase the perceived need for additional efforts (Buhrau, 2020). Hence, understanding who has dietary approach and/or avoidance intentions is highly relevant to tailor interventions. Beyond that, there is a lack of research assessing which foods are targeted as part of dietary approach and avoidance intentions, i.e., which foods do individuals intend (not) to eat. However, this information is crucial to target and personalize behaviour change interventions.

### Present study

Despite the potential of intentions in tailoring interventions, little is known about weight‐loss and dietary intentions – particularly in Germany and Austria. The present study, a large representative online survey, provides insights into (1) the prevalence of weight‐loss as well as dietary approach and avoidance intentions in Germany and Austria, (2) the strength of these intentions, (3) individual differences in these intentions and (4) the types of foods that individuals intend to approach/avoid. More specifically, this preregistered analysis (https://osf.io/unvjw) investigates the following research questions (RQ):

**RQ1:** How many people have intentions to lose weight (in total and for Germany and Austria separately)?
**RQ1a:** Do people who have vs. who do not have the intention to lose weight differ in relation to gender, age, BMI, country of residence, education and household income?
**RQ1b:** Does the strength of the intention differ in relation to gender, age, BMI, country of residence, education and household income?
**RQ2:** How many people have intentions to eat more of certain foods (in total and for Germany and Austria separately)?
**RQ2a:** Do people who have vs. do not have the intention to eat more of certain foods differ in relation to gender, age, BMI, country of residence, education and household income?
**RQ2b:** Does the strength of the intention differ in relation to gender, age, BMI, country of residence, education and household income?
**RQ2c:** The intake of which foods is intended to be increased?
**RQ3:** How many people have intentions to eat less of certain foods (in total and for Germany and Austria separately)?
**RQ3a:** Do people who have vs. do not have the intention to eat less of certain foods differ in relation to gender, age, BMI, country of residence, education and household income?
**RQ3b:** Does the strength of the intention differ in relation to gender, age, BMI, country of residence, education and household income?
**RQ3c:** The intake of which foods is intended to be reduced?

Additional to the preregistered research questions, two exploratory research questions were added that were suggested during peer review:

**RQ.e1:** Do people who have vs. who do not have a discrepancy between weight‐loss intention and weight‐loss indication differ in relation to gender, age, BMI, country of residence, education and household income?
**RQ.e2:** How frequently do specific combinations of intentions co‐occur?

## METHODS

The study sample, design, procedure and all measures (https://osf.io/s2wya) as well as the specific research questions of the present paper including planned analyses (https://osf.io/unvjw) were preregistered prior to data collection. The local ethics committee approved the study.

### Study sample

A representative sample of 1,500 adult participants (*n* = 1,000 from Germany, *n* = 500 from Austria) was aimed for. Eligible for participation were individuals aged 18 years or above, living in Germany or Austria and speaking German sufficiently well to be able to complete the survey. The sample size was determined partly based on available funding. With a sample size of 1,500, effects of Cohen's *d* = 0.15 (independent samples *t*‐test, two‐sided), Cohen's *f* = 0.09 (one‐way between‐subjects ANOVA with 5 groups, two‐sided) or w = 0.09 (χ^2^ test with *df* = 4; all α = .05, 1 – β = .80) at 80% power (c.f. G*Power 3.1, Faul et al., 2009) can be detected. Stratified samples by country based on gender, age and education using the quota shown in Appendix 1 were recruited.

### Study procedure

Data was collected as part of a representative online survey (parts of the data of this study have been used for different research questions, see study 2 in König, Kanning, et al., 2025, Harsora et al., 2025 and König, Volpi, et al., 2025). Participants were recruited through an online panel provider and asked to complete the survey on a computer. Data collection took place in May 2024. All participants provided informed consent by ticking a box after being informed about the study procedure. They then completed the survey, which included three attention check items. Participants who either failed two of the attention check items, indicated not to live in Austria or Germany, or were younger than 18 years were screened out and replaced until the pre‐registered sample size was reached.

### Measures

#### Sociodemographic information

Participants reported their age in years and were asked to indicate their gender (woman, man, gender‐diverse) and country of residence (Germany, Austria, other). Participants indicated their highest school leaving and vocational qualifications. Based on this, years of education were calculated as the sum of the years spent to obtain the highest school leaving and highest vocational qualification (see Appendix 2). Participants were asked for the monthly net income of their household based on 11 categories. The sample was then divided into income quartiles: The first quartile ranges from *less than 150€* to *1,500€ to less than 2,000€*, the second quartile from *2,000€ to less than 2,500€* to *2,500€ to less than 3,000€*, the third quartile comprises the category *3,000€ to less than 5,000€* and the fourth quartile ranges from *5,000€ to less than 10,000€* to *over 10,000€*.

#### Body mass index (BMI)

Participants self‐reported body height (in metres) and body weight (in kilograms) which were used to calculate BMI according to the following formula: weight/(height*height). To avoid incorrect entries, an error message was displayed when the height entry was below 1 m or above 3 m and when reported weight was below 10 kg or above 500 kg.

#### Intentions

##### Weight‐loss intention

The intention to lose weight was assessed using two closed questions. First, participants indicated whether they currently have the intention to lose weight (Do you currently intend to reduce your body weight? *yes*/*no*). If participants indicated that they currently intend to lose weight, the strength of the intention was assessed on a 7‐point Likert scale from *not at all* to *very*.

##### Dietary intentions

Intentions to eat more/less of certain foods (i.e., dietary approach and avoidance intentions) were assessed using two closed questions (intention: Do you currently intend to eat more/less of certain foods? *yes*/*no*; if *yes*, strength of intention: How much do you currently intend to eat more/less of these foods? 7‐point Likert scale from *not at all* to *very*) as well as one open question assessing the foods that people intend to eat more/less of if they indicated that they currently intend to eat more/less of certain foods: Which foods do you currently intend to eat more/less of?

### Data preprocessing

Participants who indicated to have an intention but rated the strength of the intention as non‐existing (1 on the 7‐point scale) were excluded from the strength analyses (weight‐loss intention: *n* = 1; dietary approach intention: *n* = 2; dietary avoidance intention: *n* = 2). Responses of the open questions assessing the target foods of the dietary approach and avoidance intentions were coded according to 19 preregistered food groups, derived from the dietary guidelines of the German Nutritional Society (Schäfer et al., 2024) and the German Food Code and Nutrient Data Base (Bundeslebensmittelschlüssel, Hartmann et al., 2008), one added food group (i.e., fast food) as well as 16 added overarching categories (see Table 1). If participants named more than one food, each food was coded individually. Foods that could not clearly be assigned to one of the categories in Table 1 (e.g., ‘Gebäck’, English: baked goods/pastries, which could either be sweet and assigned to ‘snacks high in sugar, fat, and/or salt’ or savoury and assigned to ‘cereals, bread, and pasta*’*) or that did not match any category (e.g., ‘moringa’ or ‘foods containing purine’) were coded as ‘others’. Participants who named only non‐food items (dietary approach intention: *n* = 18; dietary avoidance intention: *n* = 34) or only foods that were categorized as ‘others’ (dietary approach intention: *n* = 3; dietary avoidance intention: *n* = 8) were excluded from the respective analyses.

**TABLE 1:** Overview of food categories used to code target foods of the dietary approach and avoidance intentions.

Preregistered	Added during first coding stage	Added during discussion stage
Fruits and vegetables	Fast food	Salt/foods high in salt
Juices	Sugar/foods high in sugar	
Legumes	Fat/foods high in fat	
Nuts and seeds	Protein/foods high in protein	
Potatoes	Carbs/foods high in carbs	
Cereals, bread and pasta *(not whole‐grain)*	Fibre/foods high in fibre	
Cereals, bread and pasta *(whole‐grain)*	Vitamins/foods high in vitamins	
Vegetable oils	Organic foods	
Animal‐based fats (e.g., lard)	Healthy foods	
Dairy products	High calorie foods	
Fish	Vegan/plant‐based foods	
Meat	Unhealthy foods	
Processed meat products *(*e.g.*, [sliced] sausage)*	Animal‐based foods	
Eggs	Highly processed foods/ready meals	
Snacks high in sugar, fat and/or salt *(*e.g.*, cake, crisps)*	Foods containing gluten	
Plant‐based alternatives *(*e.g.*, plant‐based milk)*	Foods containing lactose	
Water/calorie‐free beverages *(*e.g.*, unsweetened tea)*		
Sugar‐sweetened beverages		
Alcoholic beverages		

In the first coding stage, rater 1 coded all target foods and realized that the 19 pre‐registered categories did not cover all responses. For instance, a large proportion of participants responded on the macronutrient level (e.g., foods high in sugar) instead of the food level (e.g., chocolate) or named qualitative characteristics of foods (e.g., healthy foods, plant‐based foods). Therefore, the coding system was adjusted by adding categories that occurred at least twice (see an overview of added categories in Table 1) before the second coding stage in which rater 2 coded 20% of the responses. After rater 2 completed the coding, unweighted Cohen's kappa was calculated as a measure of interrater reliability. Agreement was defined as all codes of one participant being identical across the two raters. For instance, if a participant's entry included three foods and the raters agreed on the codes of two of the foods, but not on the third, the entry was counted as disagreement. Cohen's kappa was 0.48 for target foods of approach intentions and 0.76 for target foods of avoidance intentions. In the following stage, disagreement was discussed and resolved and a last category was added (see Table 1). During the discussion it became apparent that rater 2 coded categories twice when participants named more than one food of that category. However, rater 1 only coded it once. This issue was particularly prevalent for entries including ‘fruit and vegetables’ and caused the low interrater agreement for target foods of dietary approach intentions. It was decided that each category was coded only once per participant. Another disagreement was identified for ‘Süßes’ which can either be understood as ‘something sweet’ or ‘sweets’. Rater 1 coded it as ‘snacks high in sugar, fat, and/or salt (e.g., cake, crisps)’, rater 2 as ‘sugar/ foods high in sugar’. The raters agreed to code it as ‘snacks high in sugar, fat and/or salt (e.g., cake, crisps)’, as sugar was not explicitly mentioned. It was also decided that responses which included examples were only coded as the more general category (e.g., ‘vegan, protein‐rich foods, such as chickpeas’ only coded as ‘protein /foods high in protein’ and ‘vegan/plant‐based foods’, not ‘legumes’).

### Data analysis

Analyses were conducted using R (R Core Team, 2024) and RStudio (Posit team, 2024). Data and R code are available on the OSF (https://osf.io/swupm/files/osfstorage). As preregistered, we provide descriptive statistics for the samples' (in total and by country) sociodemographic variables and BMI as well as the proportions of people indicating they have a weight‐loss intention (RQ1), dietary approach intention (RQ2) or dietary avoidance intention (RQ3) and the food group‐specific proportion of individuals intending to eat more (RQ2c) or less (RQ3c) of a food of each food category. In addition to the preregistered analyses, we looked at weight‐loss indications (i.e., meeting BMI criteria for weight loss recommendation with an BMI of 25 or above) as part of RQ1. Beyond that, we carried out two exploratory analyses which were suggested during peer review: (1) comparison across individuals who have vs. who do not have a discrepancy between weight‐loss intention and weight‐loss indication (RQ.e1) and (2) frequencies of co‐occurrences of intentions (RQ.e2).

To compare people with and without a weight‐loss intention (RQ1a), dietary approach intention (RQ2a), dietary avoidance intention (RQ3a) or a discrepancy between weight‐loss intention and weight‐loss indication (RQ.e1) in terms of gender (excluding individuals who identify as gender‐diverse due to the small sample size, *n* = 4), country of residence and household income quartiles, chi‐square tests were used. For the comparisons in terms of age, BMI and years of education, independent *t*‐tests were planned.

To compare the strength of the weight‐loss intention (RQ1b), dietary approach intention (RQ2b) or dietary avoidance intention (RQ3b) across income quartiles, one‐way between‐subjects ANOVAs were planned. Significant main effects were followed up with Bonferroni‐corrected post‐hoc tests. To compare the strength of the intentions across gender and country of residence, independent *t*‐tests were planned. To test whether age, BMI and years of education predict the strength of the intentions, a multiple linear regression model with the intention strength as the dependent variable and age, BMI and years of education as independent variables was calculated for each of the three intentions.

For all analysis, a significance level of .05 was used and effect sizes are reported and interpreted according to Cohen (1992). If the assumptions of normal distribution of the dependent variable in each group or of the residuals and homogeneity of variances for independent *t*‐tests and ANOVAs were not met, Mann–Whitney U tests (for age, BMI and education in RQ1a, RQ2a and RQ3a; for gender and country of residence in RQ1b, RQ2b and RQ3b) or Kruskal‐Wallis tests (for income quartiles in RQ1b, RQ2b and RQ3b) were used, respectively. Prior to interpreting the results of the multiple linear regression models, normality of residuals, homoscedasticity, multicollinearity and independence across observations were tested. Even when the normality assumption was violated, results were interpreted as they are expected to be still valid given the large sample size of the present study (Schmidt & Finan, 2018). In the case of autocorrelation, model coefficients with robust standard errors were used (see RQ3b).

## RESULTS

### Sample characteristics

In total, 1,510 participants completed the study. Sociodemographic characteristics of the total sample as well as the sample from Germany and Austria separately can be found in Table 2.

**TABLE 2:** Sociodemographic characteristics of the total sample as well as the German and Austrian samples.

	Total sample (*N* = 1,510)		Germany (*n* = 1,006)		Austria (*n* = 504)	
Variable	*n*/*M* (*SD*)	% /*range*	*n*/*M* (*SD*)	% /*range*	*n*/*M* (*SD*)	% /*range*
Gender						
Women	761	50.40	506	50.30	255	50.60
Men	745	49.34	496	49.30	249	49.41
Gender‐diverse	4	0.27	4	0.40	0	0
Age (years)	48.55 (16.68)	18–83	48.89 (16.67)	18–83	47.89 (16.72)	18–83
BMI^a^	26.89 (5.92)	13.15–60.04	26.93 (6.03)	16.36–60.04	26.80 (5.71)	13.15–49.54
Weight status^a^						
Underweight (BMI < 18.5)	36	2.38	22	2.19	14	2.78
Normal weight (BMI ≥ 18.5)	609	40.33	414	41.15	195	38.69
Overweight (BMI ≥ 25)	488	32.32	319	31.71	169	33.53
Obesity (BMI ≥ 30)	355	23.51	236	23.46	119	23.61
Years of education	14.15 (2.73)	8–20	13.88 (2.76)	8–20	14.68 (2.57)	8–20
Monthly net income^b^						
First quartile	492	32.58	343	34.10	149	29.56
Second quartile	402	26.62	267	26.54	135	26.79
Third quartile	463	30.66	296	29.42	167	33.14
Fourth quartile	152	10.07	99	9.84	53	10.52

^a^ BMI is missing for 22 participants.

^b^ Monthly net income missing for 1 participant from Germany.

### Weight‐loss intentions

#### RQ1: How many people have intentions to lose weight (in total and for Germany and Austria separately)?

In the total sample, 57.42% (*n* = 867) indicated to currently intend to lose weight. Proportions of the German and Austrian sample separately are shown in Table 3. Across all participants, 42.58% (*n* = 643) report having a weight‐loss intention while also having an indication for weight loss (BMI ≥ 25), 13.25% (*n* = 200) do not have a weight‐loss intention despite having an indication for weight loss, 14.04% (*n* = 212) have a weight‐loss intention despite not having a weight‐loss indication and 28.68% (*n* = 433) neither have a weight‐loss intention nor a weight‐loss indication.

**TABLE 3:** Number and proportion of people with and without a weight‐loss, dietary approach and dietary avoidance intention of the total sample as well as the German and Austrian samples.

Variable	Total sample		Germany		Austria	
	*n*	%	*n*	%	*n*	%
Weight‐loss intention						
Yes	867^a^	57.42	562^a^	55.87	305	60.52
No	643	42.58	444	44.14	199	39.48
Dietary approach intention						
Yes	520^b^	34.44	327^b^	32.51	193^b^	38.29
No	990	65.56	679	67.50	311	61.71
Dietary avoidance intention						
Yes	891^c^	59.01	573^c^	56.96	318	63.10
No	619	40.99	433	43.04	186	36.91

^a^ This includes one person who indicated having an intention but rated the strength of the intention as non‐existing (i.e., 1).

^b^ This includes two people (one from the German and one from the Austrian sample) who indicated having an intention but rated the strength of the intention as non‐existing (i.e., 1).

^c^ This includes two people from the German sample who indicated having an intention but rated the strength of the intention as non‐existing (i.e., 1).

##### RQ1a: Do people who have vs. who do not have the intention to lose weight differ in relation to gender, age, BMI, country of residence, education and household income?

Descriptives of gender, age, BMI, country of residence, years of education and household income quartiles for individuals with and without a weight‐loss intention can be found in Table 4. While the relationship between having a weight‐loss intention and gender was statistically significant, χ^2^(1, *N* = 1,506) = 3.88, *p* = .049, the strength of this association was small, Cohen's ω = 0.052 (Cohen, 1992). There was no significant relationship between having a weight‐loss intention and country of residence, χ^2^(1, *N* = 1,510) = 2.78, *p* = .095, Cohen's ω = 0.044, as well as income quartiles, χ^2^(3, *N* = 1,509) = 3.59, *p* = .309, Cohen's ω = 0.049. Mann–Whitney U tests showed no difference between people who have vs. who do not have the intention to lose weight based on age, *U* = 272,396, *Z* = −0.76, *p* = .449, *r* = −.020 and years of education, *U* = 268,084, *Z* = −1.29, *p* = .197, *r* = −.033. Individuals with weight‐loss intentions had on average a significantly higher BMI (*M* = 28.80) than individuals without weight‐loss intentions (*M* = 24.32), *U* = 130,592, *Z* = −17.09, *p* < .001, *r* = −.443.

**TABLE 4:** Descriptives of people with and without a weight‐loss, dietary approach and dietary avoidance intention.

	Weight‐loss intention		Dietary approach intention		Dietary avoidance intention	
Variable	Yes	No	Yes	No	Yes	No
	*n* = 867	*n* = 643	*n* = 520	*n* = 990	*n* = 891	*n* = 619
Gender, *n* (% of the total sample)						
Women, *n* = 761	456 (30.20)	305 (20.20)	280 (18.54)	481 (31.85)	487 (32.25)	274 (18.15)
Man, *n* = 745	408 (27.02)	337 (22.32)	238 (15.76)	507 (33.58)	402 (26.62)	343 (22.72)
Gender‐diverse, *n* = 4	3 (0.20)	1 (0.07)	2 (0.13)	2 (0.13)	2 (0.13)	2 (0.13)
Age, *M* (*SD*)	48.90 (16.14)	48.09 (17.39)	47.13 (16.66)	49.30 (16.66)	48.88 (16.43)	48.08 (17.05)
BMI^a^, *M* (*SD*)	28.79 (5.75)	24.32 (5.13)	27.11 (5.87)	26.77 (5.95)	27.81 (5.90)	25.55 (5.70)
Country of residence, *n* (% of the total sample)						
Germany, *n* = 1,006	562 (37.22)	444 (29.40)	327 (21.66)	679 (44.97)	573 (37.95)	433 (28.68)
Austrian, *n* = 504	305 (20.20)	199 (13.18)	193 (12.78)	311 (20.60)	318 (21.06)	186 (12.32)
Years of education, *M* (*SD*)	14.23 (2.68)	14.04 (2.78)	14.41 (2.74)	14.01 (2.71)	14.27 (2.68)	13.97 (2.79)
Household income^b^, *n* (% of the total sample)						
First quartile	271 (17.96)	221 (14.65)	176 (11.66)	316 (20.94)	280 (18.56)	212 (14.05)
Second quartile	238 (15.77)	164 (10.87)	145 (9.61)	257 (17.03)	237 (15.71)	165 (10.93)
Third quartile	276 (18.29)	187 (12.39)	150 (9.94)	313 (20.74)	284 (18.82)	179 (11.86)
Fourth quartile	81 (5.37)	71 (4.71)	49 (3.25)	103 (6.83)	90 (5.96)	62 (4.11)

^a^ BMI missing for 22 participants.

^b^ Monthly net income missing for 1 participant from Germany.

##### RQ1b: Does the strength of the intention differ in relation to gender, age, BMI, country of residence, education and household income?

The mean intention strength of individuals who indicated having a weight loss intention was 5.25 (*SD* = 1.21). The average strength of the weight‐loss intention was slightly stronger in individuals who identify as a woman (*M* = 5.36, *SD* = 1.29) compared to individuals who identify as a man (*M* = 5.14, *SD* = 1.10), *U* = 102,791, *Z* = −2.83, *p* = .005, *r* = −.10, and in individuals living in Austria (*M* = 5.38, *SD* = 1.19) compared to individuals living in Germany (*M* = 5.19, *SD* = 1.21), *U* = 77,721, *Z* = −2.31, *p* = .021, *r* = −.08. A Kruskal‐Wallis test showed no differences in the weight‐loss intention strength across household income quartiles, *H*(3) = 2.42, *p* = .490, η^2^ < 0.01. The results of the multiple linear regression model indicate that age (*b* = −0.006, 95% CI [−0.0108, −0.0006], *p* = .030, *f* ^*2*^ = 0.006) and BMI (*b* = 0.04, 95% CI [0.02, 0.05] *p* < .001, *f* ^*2*^ = 0.03), but not years of education (*b* = 0.002, 95% CI [−0.028, 0.033], *p* = .892, *f* ^*2*^ = 0.00002), significantly predict the strength of the weight‐loss intention.

### Dietary approach intentions

#### RQ2: How many people have intentions to eat more of certain foods (in total and for Germany and Austria separately)?

In the total sample, 34.44% of people (*n* = 520) indicated to currently intend to eat more of certain foods. Proportions in the German and Austrian sample can be found in Table 3.

##### RQ2a: Do people who have vs. do not have the intention to eat more of certain foods differ in relation to gender, age, BMI, country of residence, education and household income?

Individuals with and without dietary approach intentions are described in terms of gender, age, BMI, country of residence, years of education and household income quartiles in Table 4. Having a dietary approach intention was not associated with gender, χ^2^(1, *N* = 1,506) = 3.71, *p* = .054, Cohen's ω = 0.051 and income quartiles, χ^2^(3, *N* = 1,509) = 2.04, *p* = .564, Cohen's ω = 0.037. The relationship between having a dietary approach intention and country of residence, χ^2^(1, *N* = 1,510) = 4.73, *p* = .030, Cohen's ω = 0.058, age, *U* = 276,610, *Z* = −2.39, *p* = .017, *r* = −.061, as well as years of education, *U* = 235,469, *Z* = −2.76, *p* = .006, *r* = −.071, was significant. Yet, the effect sizes indicate that the strength of these associations is small. No significant association was found in terms of BMI, *U* = 239,172, *Z* = −1.39, *p* = .166, *r* = −.036.

##### RQ2b: Does the strength of the intention differ in relation to gender, age, BMI, country of residence, education and household income?

Individuals who have the intention to eat more of certain foods had a mean approach intention strength of 5.83 (*SD* = 1.05). The average strength of the dietary approach intention was slightly stronger in individuals who identify as a woman (*M* = 5.95, *SD* = 1.02) compared to individuals who identify as a man (*M* = 5.69, *SD* = 1.06), *U* = 37,524, *Z* = −2.76, *p* = .006, *r* = −.12. Individuals living in Austria (*M* = 5.80, *SD* = 1.09) did not differ from individuals living in Germany (*M* = 5.84, *SD* = 1.02) in the strength of their dietary approach intention, *U* = 31,530, *Z* = −0.15, *p* = .882, *r* = −.007. Neither age (*b* = 0.005, 95% CI [−0.001, 0.011], *p* = .086, *f* ^*2*^ = 0.006), BMI (*b* = 0.008, 95% CI [−0.008, 0.024], *p* = .354, *f* ^*2*^ = 0.002), nor years of education (*b* = −0.006, 95% CI [−0.039, 0.028], *p* = .740, *f* ^*2*^ = 0.0002) significantly predict the strength of the dietary approach intention. No difference in the dietary approach intention strength was found across household income quartiles, *H*(3) = 0.74, *p* = .863, η^2^ < 0.01.

##### RQ2c: The intake of which foods is intended to be increased?

The proportion of people (*n*, %) intending to eat more of a food of each food category is shown in Table 5 for the complete sample. The same table for Germany and Austria separately can be found in Appendix 3. Across both countries, the most frequently named food group was ‘fruit and vegetables’ (27.09%), followed by ‘protein/foods high in protein’ (2.65%).

**TABLE 5:** Overview of how many participants named at least one food of each category as a target food of their dietary approach or avoidance intention.

	Dietary approach intention	Dietary avoidance intention
**Food groups**, *n* (% of the total sample)		
Fruit and vegetables	409 (27.09)	5 (0.33)
Juices	0	2 (0.13)
Legumes	5 (0.33)	0
Nuts and seeds	7 (0.46)	2 (0.13)
Potatoes	4 (0.27)	9 (0.60)
Cereals, bread and pasta *(not whole‐grain)*	13 (0.86)	55 (3.64)
Cereals, bread and pasta *(whole‐grain)*	11 (0.73)	0
Vegetable oils	1 (0.07)	1 (0.07)
Animal‐based fats (e.g., lard)	0	5 (0.33)
Dairy products	12 (0.80)	12 (0.8)
Fish	15 (0.99)	0
Meat	21 (1.39)	176 (11.66)
Processed meat products *(*e.g.*, [sliced] sausage)*	0	24 (1.59)
Eggs	3 (0.20)	1 (0.07)
Snacks high in sugar, fat and/or salt *(*e.g.*, cake, crisps)*	1 (0.07)	364 (24.11)
Plant‐based alternatives *(*e.g.*, plant‐based milk)*	0	0
Water/calorie‐free beverages *(*e.g.*, unsweetened tea)*	3 (0.20)	0
Sugar‐sweetened beverages	1 (0.07)	8 (0.53)
Alcoholic beverages	0	18 (1.19)
Fast food	0	42 (2.78)
**Additional categories**, *n* (% of the total sample)		
Sugar/foods high in sugar	1 (0.07)	171 (11.33)
Fat/foods high in fat	0	94 (6.23)
Protein/foods high in protein	40 (2.65)	0
Carbs/foods high in carbs	0	84 (5.56)
Fibre/foods high in fibre	7 (0.46)	0
Vitamins/foods high in vitamins	2 (0.13)	0
Organic foods	5 (0.33)	0
Healthy foods	7 (0.46)	0
High calorie foods	3 (0.2)	11 (0.73)
Vegan/plant‐based foods	6 (0.40)	0
Unhealthy foods	0	8 (0.53)
Animal‐based foods	0	8 (0.53)
Highly processed foods/ready meals	0	18 (1.19)
Foods containing gluten	0	3 (0.20)
Foods containing lactose	0	3 (0.20)
Salt/foods high in salt	0	6 (0.40)

### Dietary avoidance intentions

#### RQ3: How many people have intentions to eat less of certain foods (in total and for Germany and Austria separately)?

More than half of the total sample (59.01%, *n* = 891) indicated to currently intend to decrease the intake of certain foods. Proportions of the German and Austrian samples separately are displayed in Table 3.

##### RQ3a: Do people who have vs. do not have the intention to eat less of certain foods differ in relation to gender, age, BMI, country of residence, education and household income?

Descriptive statistics of gender, age, BMI, country of residence, years of education and household income quartiles for individuals with and without dietary avoidance intentions are presented in Table 4. There was a significant association between having a dietary avoidance intention and gender, χ^2^(1, *N* = 1,506) = 15.26, *p* < .001, with a small effect size, Cohen's ω = 0.102. Age, *U* = 268,490, *Z* = −0.87, *p* = .383, *r* = −.022, years of education, *U* = 259,816, *Z* = −1.94, *p* = .052, *r* = −.050 and income, χ^2^(3, *N* = 1,509) = 1.94, *p* = .585, Cohen's ω = 0.036, were not significantly related to having a dietary avoidance intention. A significant relation was found between having a dietary avoidance intention and BMI, *U* = 197,139, *Z* = −8.64, *p* < .001, as well as country of residence, χ^2^(1, *N* = 1,510) = 4.98, *p* = .026. While the effect size for country of residence was very small, Cohen's ω = 0.059, the effect size for BMI was small to medium, *r* = −.224.

##### RQ3b: Does the strength of the intention differ in relation to gender, age, BMI, country of residence, education and household income?

The intention of participants to eat less of certain foods had a mean strength of 5.41 (*SD* = 1.15). Individuals who identify as a woman (*M* = 5.51, *SD* = 1.14) showed slightly stronger dietary avoidance intentions compared to individuals who identify as a man (*M* = 5.28, *SD* = 1.14), *U* = 107,129, *Z* = −2.65, *p* = .008, *r* = −.09. No difference in the strength of the dietary avoidance intention was found between individuals living in Austria (*M* = 5.39, *SD* = 1.15) and individuals living in Germany (*M* = 5.42, *SD* = 1.15), *U* = 92,391, *Z* = −0.45, *p* = .651, *r* = −.02. A multiple linear regression model with robust standard errors indicated that BMI (*b* = 0.02, 95% CI [0.001, 0.03], *p* = .036, *f* ^*2*^ = 0.006), but not age (*b* = 0.0002, 95% CI [−0.005, 0.005], *p* = .94, *f* ^*2*^ = 0.000006) and years of education (*b* = −0.02, 95% CI [−0.05, 0.01], *p* = .122, *f* ^*2*^ = 0.003), significantly predicts the strength of the dietary avoidance intention. A Kruskal‐Wallis test found no differences in the strength of the dietary avoidance intention across household income quartiles, *H*(3) = 0.94, *p* = .817, η^2^ < 0.01.

##### RQ3c: the intake of which foods is intended to be reduced?

The number and percentage of people intending to eat less of foods of each category is shown in Table 4. The same table, separately for Germany and Austria, can be found in Appendix 3. Across both countries, the most frequently named food group was ‘snacks high in sugar, fat, and/or salt’ (24.11%), followed by ‘meat’ (11.66%) and ‘sugar/foods high in sugar’ (11.33%).

### Exploratory analyses

#### RQ.e1: Do people who have vs. who do not have a discrepancy between weight‐loss intention and weight‐loss indication differ in relation to gender, age, BMI, country of residence, education and household income?

Descriptives of gender, age, BMI, country of residence, years of education and household income quartiles for individuals with and without a discrepancy between weight‐loss intention and weight‐loss indication can be found in Table 6. Individuals with and without a discrepancy between weight‐loss intention and weight‐loss indication did not differ in terms of gender, χ^2^(1, *N* = 1,484) = 0.35, *p* = .55, Cohen's ω = 0.017, country of residency, χ^2^(1, *N* = 1,488) = 0.05, *p* = .82, Cohen's ω = 0.008, income, χ^2^(3, *N* = 1,487) = 1.25, *p* = .74, Cohen's ω = 0.029 and years of education, *U* = 228,062, *Z* = −0.88, *p* = .38, *r* = −.02. A significant difference was found for age, *U* = 236,646, *Z* = −2.02, *p* = .04, *r* = −.05, and BMI, *U* = 244,909, *Z* = −3.14, *p* = .002, *r* = −.08. However, effect sizes were very small.

**TABLE 6:** Descriptives of individuals with and without a discrepancy between weight‐loss intention and weight‐loss indication.

	No discrepancy between weight‐loss intention & weight‐loss indication (*n* = 1,076, 72.31%)	Discrepancy between weight‐loss intention & weight‐loss indication (*n* = 412, 27.69%)
Variable	*n (%)* /*M* (*SD*)	*n* (%) /*M* (*SD*)
Gender		
Women	535 (35.95%)	212 (14.25%)
Men	539 (36.22%)	198 (13.31%)
Gender‐diverse	2 (0.13%)	2 (0.13%)
Age (years)	49.12 (16.45)	47.09 (17.37)
BMI	27.16 (6.12)	26.17 (5.31)
Country of residence		
Germany	719 (48.32%)	272 (18.28%)
Austria	357 (23.99%)	140 (9.41%)
Years of education	14.19 (2.74)	14.02 (2.70)
Monthly net income		
First quartile	344 (23.13%)	140 (9.42%)
Second quartile	286 (19.23%)	112 (7.53%)
Third quartile	334 (22.46%)	124 (8.34%)
Fourth quartile	111 (7.47%)	36 (2.42%)

*Note*: This table only includes participants for whom BMI is available (*n* = 1,488).

#### RQ.e2: How frequently do specific combinations of intentions co‐occur?

**FIGURE 1:**
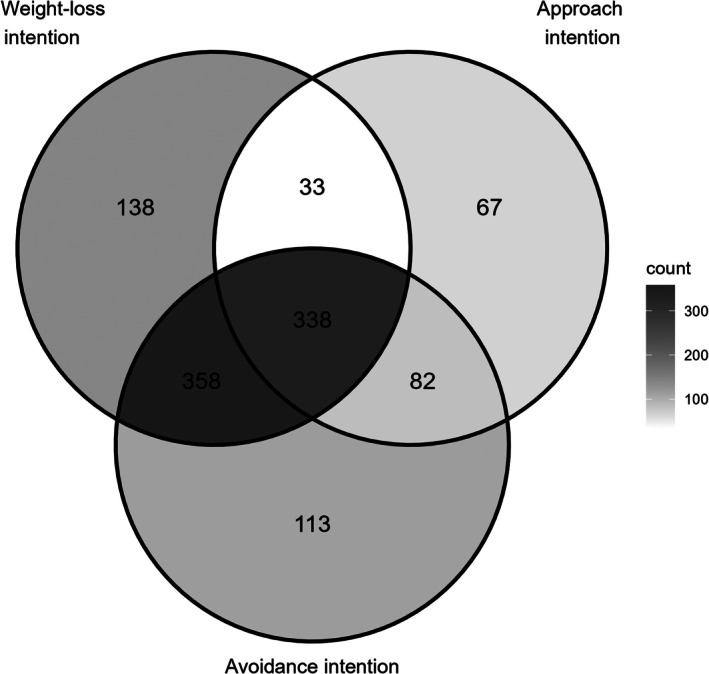
Venn diagram displaying co‐occurrence of weight‐loss, dietary approach and dietary avoidance intentions.

In total, 381 participants (25.23%) reported not having any of the three intentions. The frequency of combinations of co‐occurring intentions can be found in Figure 1. The most common co‐occurrences were (1) weight‐loss and dietary avoidance intention (*n* = 358, 23.71%) and (2) all three intentions (*n* = 338, 22.38%).

## DISCUSSION

Given the importance of intentions for behaviour change and the potential increased effectiveness of interventions when tailored to specific goals (König et al., 2021), the present study explored weight‐loss and dietary intentions in the German and Austrian population. Results show that over half of the sample has a weight‐loss and dietary avoidance intention, but only about one third of the sample has a dietary approach intention. While a quarter of the sample reported not having any of the three intentions, weight‐loss and dietary avoidance intentions co‐occurred in almost 24% of participants and almost 23% reported to have all three intentions. Participants most frequently indicated to intend to reduce the consumption of ‘snacks high in sugar, fat, and/or salt’, ‘meat’ and ‘sugar/ foods high in sugar’, and most frequently indicated to intend to increase the consumption of ‘fruits and vegetables’ and ‘protein/foods high in protein’, although much less frequent. While some socio‐demographic characteristics were associated with having weight‐loss and/or dietary intentions and/or their strengths, effect sizes were rather small, except for BMI and having a weight‐loss intention: Individuals with weight‐loss intentions had a significant higher BMI than individuals without weight‐loss intentions.

The prevalence of weight‐loss intentions in Germany (56%) and Austria (61%) is slightly lower compared to the prevalences of adults in the US (2015–2016: 67%) reported by Han et al. (2019). Interestingly, the increasing trend in the prevalence of weight‐loss intentions among adults in the US from 62% in 1999–2000 to 67% in 2015–2016 was accompanied by increases in BMI over the same period of time, but not an increase in the prevalence of considering oneself as having overweight (Han et al., 2019). Similarly, while the prevalence of weight‐loss intentions in the present sample (57%) is close to the overall prevalence of overweight and obesity (56%), not all individuals who have an indication for weight loss report having a weight‐loss intention and vice versa. This discrepancy between weight‐loss indication and intention was also found in Lee et al. (2004) – only 38% of individuals with an indication to lose weight were trying to lose weight, while 17% of individuals were trying to lose weight despite not having an indication for weight loss. In the present sample, 13% reported not having a weight‐loss intention despite having an indication for weight loss, while 14% reported a weight‐loss intention despite not having a weight‐loss indication. However overall, individuals with weight‐loss intentions had a significantly higher BMI than individuals without weight‐loss intentions. This is in line with (1) Han et al. (2019) who report lower prevalences of the intention to weigh less in individuals with normal weight (BMI < 25; 2015–2016: 28%) compared to individuals with overweight (BMI ≥ 25; 2015–2016: 82%) and (2) Lee et al. (2004) who found that adults aged 70 to 79 years with weight‐loss intentions were more likely to have higher body weight. Hence, while individuals with weight‐loss intentions report/have higher BMIs overall, the discrepancy between weight‐loss intention and weight‐loss indication in over one quarter of the sample highlights the need to raise awareness of who does (not) need to (intend to) lose weight. Specifically, younger individuals and individuals with lower BMI were more likely to have a discrepancy between weight‐loss intention and weight‐loss indication. This highlights the importance of tailored risk communication and behaviour change efforts to ensure adequate intention formation. Yet, the present study did not examine reasons for weight loss. Although overweight and particularly obesity are related to a range of negative health consequences, it is important to note that increased body weight does not always lead to worse health outcomes (Rubino et al., 2025). Given that risk perception and outcome expectancies are important predictors of intentions (Schwarzer, 1992, 2008), one could assume that participants without a weight‐loss intention are actually, or at least perceive themselves as not at risk and therefore see no need to act. Beyond that, the present study did not assess intended strategies for weight loss. It is important to highlight that, while dietary approach and avoidance intentions are linked to one specific behaviour, weight‐loss intentions may include multiple health behaviours (e.g., eating behaviour, physical activity). Future studies therefore may need to extend the present analysis by also including physical activity goals.

Similar to Houle‐Johnson and Kakinami (2018) and Lee et al. (2004), the present study found that individuals who identify as a woman are more likely to have weight‐loss intentions, and their weight‐loss intentions are stronger compared to individuals who identify as a man. Reasons for this could be gender‐specific weight ideals. For instance, in relation to BMI, a study by Crossley et al. (2012) found that the female ideal is associated with a relatively low BMI (i.e., of about 18.8), while the male ideal is associated with a higher BMI (i.e., of about 25). However, it is important to highlight that the effects of gender in the present study were only small – which was also the case for associations found between weight‐loss intention strength and age, BMI and country of residence: The strength of the weight‐loss intention was stronger for individuals who identify as a woman, who live in Austria, who are younger and who have a higher BMI. Contrary to Lee et al. (2004) who found that individuals with weight‐loss intentions were more likely to have graduated from high school, no relation was found between weight‐loss intentions and their strengths and education as well as income.

Interestingly, a comparison of the prevalence of dietary approach and avoidance intentions shows that the prevalence of dietary avoidance intentions (59%) is considerably higher than the prevalence of dietary approach intentions (34%). This indicates that individuals might focus predominantly on avoiding 'unhealthy' foods rather than on approaching 'healthy' foods. Especially when individuals are intending to lose weight, avoidance intentions seem particularly prevalent, as shown by the high co‐occurrence of weight‐loss and dietary avoidance intentions. This might be problematic, as research suggests that approach strategies might be more effective in improving diet quality (David & Haws, 2016). In line with this, implementation intentions have been found to be more effective in promoting healthy eating than in reducing unhealthy eating (Adriaanse et al., 2011; Carrero et al., 2019). Yet, a study highlights that the stage of goal pursuit should be considered when choosing between approach and avoidance strategies, such that individuals who are far from goal attainment benefit most from approach strategies, while avoidance strategies are more effective in individuals who are close to their goal (Buhrau, 2020). Hence, individual‐focused interventions could track goal progress of the behaviour‐related outcome (e.g., weight status) to tailor provided strategies (i.e., switching from approach to avoidance strategies when individuals get closer to their goal). However, population‐wide initiatives might have the biggest impact implementing approach strategies, as they are more likely to be beneficial to individuals who are at highest risk. While effective strategies are available for approach behaviours, for instance, fruit and vegetable consumption (Wolfenden et al., 2021), more research is needed to understand why avoidance intentions are almost twice as prevalent compared to approach intentions, i.e., whether this tendency is rooted in psychological, social and/or cultural factors. Furthermore, given the imbalance, the feasibility of promoting approach strategies needs to be carefully evaluated. In this context, it is also important to note that, despite the focus of the present study on individual‐level factors, intentions are shaped by a variety of factors, including contextual and temporal factors. Future studies are needed to better understand how these factors influence intention formation.

Taking a closer look at the target foods of dietary intentions shows that the most common target foods of dietary approach and avoidance intentions are in line with dietary recommendations. For instance, the German Nutrition Society (DGE) recommends avoiding sweet, salty and fatty foods and limiting meat and sausage intake (German Nutrition Society, 2024) which corresponds with the three most common target foods of dietary avoidance intentions: ‘snacks high in sugar, fat, and/or salt’, ‘meat’ and ‘sugar/foods high in sugar’. Also the most prevalent target foods of dietary approach intentions ‘fruits and vegetables’ align with the DGE's recommendation to eat at least five portions of fruit and vegetables per day (German Nutrition Society, 2024). This indicates that individuals living in Germany and Austria are at least somewhat familiar with dietary recommendations. However, studies show that recommendations are often not met. For instance, a study shows that only 45.1% of women and 24.1% of men report to eat fruit and vegetables daily in Germany (Richter et al., 2021). Hence, tailored interventions are needed to support individuals in translating their intentions into behaviour. While tailoring interventions based on intentions can be useful to support their translation into behaviour, it is important to highlight that not every intention is a good intention. For instance, losing weight is typically recommended only for individuals with BMIs of 25 or above. Yet, as mentioned above, 13% of participants indicated to have a weight‐loss intention despite having a BMI below 25. Since reasons for weight loss were not assessed, we cannot differentiate between people intending to lose weight for health reasons despite having an BMI below 25 (i.e., not knowing what the recommendation is), and people who want to lose weight due to other reasons (e.g., body appearance/image, social norms) despite knowing that weight loss is generally not recommended for them from a health perspective. Hence, there also seems to be a need for interventions that identify and change maladaptive intentions and thereby align people's intentions with general weight/health recommendations.

While the study has some strengths, such as the large, representative samples from two countries, some limitations need to be considered: (1) Body weight and height were self‐reported, which might produce bias. For instance, a systematic review found that individuals who identify as a woman might underreport their weight, while individuals who identify as a man might overreport their height (Fayyaz et al., 2024), causing biased BMI estimates. (2) No information on an individual's dietary intake was collected. Therefore, no conclusions can be drawn about whether the reported dietary approach and avoidance intentions comply with general dietary recommendations. Since weight‐loss intentions seem to not always correspond to the actual need of the individual, this might also be the case for dietary approach and avoidance intentions. For instance, it is possible that individuals might have the intention to eat more fruit and vegetables, despite already consuming the recommended amounts or individuals might not have the intention to eat more fruit and vegetables, despite not consuming the recommended amounts. Alternatively, a ‘true’ intention‐behaviour gap might be present in such a way that individuals might intend to eat more fruit and vegetables but still not reach the recommended amounts. Yet, it is also possible that individuals might not intend to eat more fruit and vegetables, as they already consume the recommended amounts. Given that the present study lacks information on actual dietary intake, it is not possible to differentiate between these four cases. Hence, future studies are needed to better understand the relationship between intentions and behaviour. (3) Although several analyses yielded statistically significant results, it is important to highlight that in large samples, statistical significance does not imply practical relevance. Since most effects can be classified as small (c.f. Cohen, 1992), their practical implications need to be assessed with caution.

## CONCLUSIONS

The present study indicates that most people have weight‐loss and dietary avoidance intentions, while dietary approach intentions are less common. Given that dietary approach intentions might be more effective as targets for interventions (e.g., implementation intentions), it is possible that individuals could benefit from a less avoidance‐centered framing of eating, that is, highlighting the potential of consuming healthy foods instead of avoiding unhealthy foods. Beyond that, the study found a discrepancy between weight‐loss intentions and weight‐loss indications. Hence, there is a need to help people recognize for whom weight loss is (not) recommended.

## CONFLICT OF INTEREST STATEMENT

The authors declare that they have no competing interests.

## ETHICS STATEMENT

The ethics committee of the University of Vienna approved the study (approval number 01141).

## Supporting information


**Data S1.** *Quota used to stratify samples by country based on gender, age and education*.

**Data S2.** *Transformation of highest school leaving qualification and highest vocational qualification into years of education*.

**Data S3.** *Overview of target foods of dietary approach and avoidance intentions in the German and Austrian samples separately*.

## Data Availability

The data and code that support the findings of this study are openly available in the Open Science Framework at https://osf.io/swupm/files.
