# Supplementary material for: What (not) to eat: Exploring weight‐loss and dietary intentions in representative samples from Germany and Austria
Source: Appl Psychol Health Well Being. 2025 Sep 13;17(5):e70077. doi: 10.1111/aphw.70077 (PMC12432808; doi:10.1111/aphw.70077)
Supplement: Supplementary file 1 — Data S1. Quota used to stratify samples by country based on gender, age and education. [file APHW-17-0-s003.pdf]

Supplement 1

Quota used to stratify samples by country based on gender, age, and education

Austria

Gender (Bundeskanzleramt, 2021)

Gender	%	*n*
Men	50.80	254
Women	49.20	246

Age (Statistik Austria, 2024)

Age group	%	*n*
18-24	9.12	46
25-34	16.30	82
35-44	16.13	81
45-54	17.30	86
55-64	17.63	88
65+	23.52	118

Education (OECD, 2021)

Category	%	*n*
Below upper secondary	14.05	70
Upper secondary	50.39	252
Tertiary	35.55	178

Germany

Gender (Statistisches Bundesamt, 2023)

Gender	%	*n*
Men	49.29	493
Women	50.71	507

Age (Statistisches Bundesamt, 2022)

**Age group**	**%**	***n***
18-24	10.33	103
25-34	15.06	151
35-44	14.32	143
45-54	17.70	177
55-64	17.18	172
65+	25.41	254

Education (OECD, 2021)

**Category**	**%**	***n***
Below upper secondary	16.46	165
Upper secondary	51.03	510
Tertiary	32.52	325

References

Bundeskanzleramt (2021). *Frauen und Männer in Österreich.* https://www.bundeskanzleramt.gv.at/dam/jcr:b99bab2b-28a9-4b7d-9e8e-89e87e17052d/gender_index_2021.pdf

OECD (2021). *Adult education level (indicator)*. https://data.oecd.org/eduatt/adult-education-level.htm#indicator-chart

Statistik Austria (2024). *Bevölkerung nach Alter/Geschlecht*. Retrieved 26 February 2024 from https://www.statistik.at/statistiken/bevoelkerung-und-soziales/bevoelkerung/bevoelkerungsstand/bevoelkerung-nach-alter/geschlecht

Statistisches Bundesamt. (2022). *Bevölkerung: Deutschland, Stichtag, Altersjahre*. https://data.europa.eu/data/datasets/30303031-3234-4031-312d-303030350000?locale=de

Statistisches Bundesamt. (2023). *Bevölkerung nach Nationalität und Geschlecht (Quartalszahlen)*. Retrieved 26 February 2024 from https://www.destatis.de/DE/Themen/Gesellschaft-Umwelt/Bevoelkerung/Bevoelkerungsstand/Tabellen/liste-zensus-geschlecht-staatsangehoerigkeit.html#651186
