# Supplementary material for: What (not) to eat: Exploring weight‐loss and dietary intentions in representative samples from Germany and Austria
Source: Appl Psychol Health Well Being. 2025 Sep 13;17(5):e70077. doi: 10.1111/aphw.70077 (PMC12432808; doi:10.1111/aphw.70077)
Supplement: Supplementary file 2 — Data S2. Transformation of highest school leaving qualification and highest vocational qualification into years of education. [file APHW-17-0-s001.pdf]

Supplement 2

Transformation of highest school leaving qualification and highest vocational qualification into years of education

Item (German)	English translation	Code	Recoded into years
**Highest school leaving qualification**			
(Noch) keinen Schulabschluss	Not (yet) a school-leaving certificate	1	8
Volksschul-, Hauptschulabschluss	Elementary school, lower secondary school	2	9
Mittlere Reife, Realschulabschluss	Intermediate secondary school leaving certificate	3	10
Abschluss der Polytechnischen Oberschule	Completion of polytechnic secondary school	4	11
Abschluss einer (Berufs-) Fachschule	Graduation from a (vocational) technical school	5	12
Hochschulreife (Abitur/ Matura)	High-school diploma (Abitur/ Matura)	6	13
Anderer Abschluss, und zwar:	Other qualification, namely:	7	11
**Highest vocational qualification**			
(Noch) keine abgeschlossene Berufsausbildung	Not (yet) a completed vocational training	1	0
Lehre	Apprenticeship	2	3
Fachoberschule/ Berufsfachschule	Technical/ vocational secondary school	3	3
Fachhochschule	Degree from a university of applied science	4	4
Hochschule	University degree	5	5
Promotion	PhD/ doctorate	6	7
Anderer Abschluss, und zwar:	Other degree, namely:	7	3
