# Supplementary material for: What (not) to eat: Exploring weight‐loss and dietary intentions in representative samples from Germany and Austria
Source: Appl Psychol Health Well Being. 2025 Sep 13;17(5):e70077. doi: 10.1111/aphw.70077 (PMC12432808; doi:10.1111/aphw.70077)
Supplement: Supplementary file 3 — Data S3. Overview of target foods of dietary approach and avoidance intentions in the German and Austrian samples separately. [file APHW-17-0-s002.pdf]

Supplement 3

Overview of target foods of dietary approach and avoidance intentions in the German and Austrian samples separately

Supplementary Table 1

Overview of how many participants named at least one food of each category as a target food of their dietary approach or avoidance intentions in the German sample

	**Dietary approach intention**	**Dietary avoidance intention**
**Food groups**, *n* (% of the German sample)		
Fruits and vegetables	252 (25.05)	1 (0.10)
Juices	0	2 (0.20)
Legumes	2 (0.20)	0
Nuts and seeds	6 (0.60)	0
Potatoes	3 (0.30)	7 (0.70)
Cereals, bread, and pasta *(not whole-grain)*	8 (0.80)	39 (3.88)
Cereals, bread, and pasta *(whole-grain)*	8 (0.80)	0
Vegetable oils	1 (0.10)	1 (0.10)
Animal-based fats (e.g., lard)	0	4 (0.40)
Dairy products	9 (0.90)	7 (0.70)
Fish	10 (0.99)	0
Meat	12 (1.19)	110 (10.93)
Processed meat products *(e.g., [sliced] sausage)*	0	16 (1.59)
Eggs	2 (0.20)	1 (0.10)
Snacks high in sugar, fat, and/or salt *(e.g., cake, crisps)*	1 (0.10)	219 (21.77)
Plant-based alternatives *(e.g., plant-based milk)*	0	0
Water/calorie-free beverages *(e.g., unsweetened tea)*	2 (0.20)	0
Sugar-sweetened beverages	0	5 (0.50)

Alcoholic beverages	0	9 (0.90)
Fast food	0	24 (2.39)
**Additional categories**, *n* (% of the German sample)		
sugar / foods high in sugar	1 (0.10)	120 (11.93)
fat / foods high in fat	0	66 (6.56)
protein / foods high in protein	29 (2.88)	0
carbs / foods high in carbs	0	55 (5.47)
fibre / foods high in fibre	6 (0.60)	0
vitamins / foods high in vitamins	1 (0.10)	0
organic foods	3 (0.30)	0
healthy foods	5 (0.50)	0
high calorie foods	3 (0.30)	9 (0.90)
vegan / plant-based foods	3 (0.30)	0
unhealthy foods	0	6 (0.60)
animal-based foods	0	3 (0.30)
highly processed foods / ready meals	0	11 (1.09)
foods containing gluten	0	2 (0.20)
foods containing lactose	0	2 (0.20)
salt / foods high in salt	0	4 (0.40)

Supplementary Table 2

Overview of how many participants named at least one food of each category as a target food of their dietary approach or avoidance intentions in the Austrian sample

	Dietary approach intention	Dietary avoidance intention
**Food groups**, *n* (% of the Austrian sample)		
Fruits and vegetables	157 (31.15)	4 (0.79)
Juices	0	0
Legumes	3 (0.60)	0
Nuts and seeds	1 (0.20)	2 (0.40)
Potatoes	1 (0.20)	2 (0.40)
Cereals, bread, and pasta *(not whole-grain)*	5 (0.99)	16 (3.18)
Cereals, bread, and pasta *(whole-grain)*	3 (0.60)	0
Vegetable oils	0	0
Animal-based fats (e.g., lard)	0	1 (0.20)
Dairy products	3 (0.60)	5 (0.99)
Fish	5 (0.99)	0
Meat	9 (1.79)	66 (13.10)
Processed meat products *(e.g., [sliced] sausage)*	0	8 (1.59)
Eggs	1 (0.20)	0
Snacks high in sugar, fat, and/or salt *(e.g., cake, crisps)*	0	145 (28.77)
Plant-based alternatives *(e.g., plant-based milk)*	0	0
Water/calorie-free beverages *(e.g., unsweetened tea)*	1 (0.20)	0
Sugar-sweetened beverages	1 (0.20)	3 (0.60)
Alcoholic beverages	0	9 (1.79)
Fast food	0	18 (3.57)
**Additional categories**, *n* (% of the Austrian sample)		
sugar / foods high in sugar	0	51 (10.12)

fat / foods high in fat	0	28 (5.56)
protein / foods high in protein	11 (2.18)	0
carbs / foods high in carbs	0	29 (5.75)
fibre / foods high in fibre	1 (0.20)	0
vitamins / foods high in vitamins	1 (0.20)	0
organic foods	2 (0.40)	0
healthy foods	2 (0.40)	0
high calorie foods	0	2 (0.40)
vegan / plant-based foods	3 (0.60)	0
unhealthy foods	0	2 (0.40)
animal-based foods	0	5 (0.99)
highly processed foods / ready meals	0	7 (1.39)
foods containing gluten	0	1 (0.20)
foods containing lactose	0	1 (0.20)
salt / foods high in salt	0	2 (0.40)
